# The Role of Public Health Services in Reducing Maternal and Newborn Health Inequalities in Urban India: A Survey Analysis of 200,000 Births Over Two Decades

**DOI:** 10.1007/s11524-026-01062-6

**Published:** 2026-06-11

**Authors:** Andrea K. Blanchard, Banadakoppa Manjappa Ramesh, Usha Ram, Prakash Kumar, Kerry Scott, James F. Blanchard, Ties Boerma

**Affiliations:** 1 Institute for Global Public Health, University of Manitoba, 771 McDermot Avenue, Winnipeg, MB R3E 0T6 Canada; 2 IGP Health Initiatives Pvt Ltd, Innov8 Coworking Space, Pride Plaza Hotel, Asset 5-A, Aerocity, Delhi, New Delhi 110037 India; 3 School of Global Health, York University, 4700 Keele Street, Toronto, ON M3J 1P3 Canada

**Keywords:** Urban health, Health inequalities, Maternal and newborn health, Public health system, India, Urban poor, Neonatal mortality

## Abstract

India has experienced rapid urbanisation, straining the healthcare system. The National Urban Health Mission was launched in 2013 to improve access to public healthcare, particularly among socio-economically disadvantaged urban populations. This study aimed to assess whether inequalities in maternal and newborn health (MNH) service coverage and outcomes between richer and poorer groups have improved at public and private sources across urban India in the last two decades. We used pooled data from four national cross-sectional surveys, the District Level Household Surveys from 2002 to 2008 and National Family Health Surveys from 2015 to 2021, covering 94,826 and 108,152 births in urban India, respectively. We analysed trends in coverage of antenatal, delivery, and postnatal care services and neonatal mortality by source across wealth deciles, and summarised inequalities using the slope index of inequality, concentration index, and inequality pattern index. The study found that coverage of all MNH services, and to a lesser extent neonatal survival, increased substantially between 2002–2008 and 2015–2021 in urban India. Improvements were steeper among the poorest groups. Coverage by public health facilities notably increased, and neonatal mortality rates were lower at public than private facilities, particularly among the poorest. However, the poorest decile remained well behind all other groups, reflecting bottom inequalities. Rapid improvements with reduced inequalities in MNH service coverage appear to be driven by increased access to public sector services in urban India. It remains critical for the public healthcare system to understand and address the particular needs of the poorest groups to reduce ongoing bottom MNH inequalities in urban India.

**Supplementary Information:**

The online version contains supplementary material available at 10.1007/s11524-026-01062-6.

## Introduction

India’s urban population has grown substantially at a rate of 2.8% per year, from 286 million in 2001 to 377 million in 2011 [1], and was estimated to reach 508 million in 2022 [2]. During the same period, the urban total fertility rate (TFR) declined from 2.3 to 1.6 children per woman, yet the absolute number of births increased from 5.8 to 7.4 million (authors’ computation based on SRS estimates of CBR [3] and projected urban population [4]). The United Nations projects that if urbanisation continues at the present rate, 40% of India’s population will be living in urban areas by 2030 [5]. This is largely due to migration into cities and rapid expansion of city boundaries, with a resultant rise in slum populations and urban poverty. According to the last census in 2011, the slum population in India was already 65 million or 17% of urban areas in India [1].

To address the unique challenges of providing comprehensive healthcare to urban populations in India’s cities, with a focus on the urban poor, vulnerable, and marginalised groups, the National Urban Health Mission (NUHM) was launched by the Government of India in 2013 as a sub-mission of the National Health Mission (NHM) [6, 7]. It sought to ensure equitable access to quality healthcare services by employing innovative strategies and strengthening convergence with diverse stakeholders and implementers to enhance the overall health and well-being of people in urban areas [6, 7]. The revised NUHM Framework 2023 underscores the government’s ongoing prioritisation of urban health, providing detailed implementation strategies, additional categories for cities of diverse sizes and types to synchronise with that of the Ministry of Housing and Urban Affairs and Census of 2011, while retaining a focus on reaching the urban poor, vulnerable, and marginalised populations with health services to reduce urban health inequities [6, 8].

A key component of the NHM has been on improving equitable access to quality healthcare during the pregnancy, childbirth, and newborn periods. Some studies in urban areas of India have assessed maternal, newborn, and child health (MNCH) indicators and inequalities in health and healthcare generally [9–11]. One analysed maternal health within a slum population [12], a few looked at wealth-based inequalities in child health in urban areas [13–15], and two others used a past national household survey to examine the source of inequalities in maternal and newborn health (MNH) intervention coverage [16, 17]. Researchers have highlighted the growing pluralism of urban health services across public and private sectors in India [18]. Relatedly, studies have examined who has benefited from conditional cash transfers and insurance schemes and who has borne high out-of-pocket expenditures for urban public or private MNH services [19–21]. Finally, many discuss the broad national policy needs in relation to the NUHM and its emphasis on reaching the most vulnerable groups with public health services [10, 22–25].

To contribute to a growing body of urban health research in India, it is relevant to analyse trends in socio-economic inequalities in MNH service coverage and survival using large-scale population representative data in urban India. With over a decade since the launch of NUHM and the recent revised NUHM 2023 guidelines, there is a need to not only examine the state of MNH inequalities but also the role of public health facilities in serving the most disadvantaged urban populations. Hence, this paper aims to systematically study trends and inequalities in neonatal mortality and coverage of critical MNH interventions by wealth decile and by source in urban India over the past two decades.

## Data and Methods

Our analysis used data from urban samples in two nationally representative sample surveys available in the public domain: the District Level Household Survey (DLHS) and the National Family Health Survey (NFHS). To overcome sample size constraints particularly for using wealth deciles, we pooled DLHS 2002–2004 (round 2) and 2007–2008 (round 3) to provide a baseline and NFHS 2015–2016 (round 4) and 2019–2021 (round 5) to provide an endline [26].

We analysed the following seven coverage indicators at baseline and endline: any antenatal care (ANC) from a skilled provider; early ANC in the first trimester; four or more ANC visits; ANC contents (with seven sub-indicators: measurement of weight and blood pressure, blood and urine samples taken, abdominal examination, Tetanus Toxoid (TT) injection, and Iron and Folic Acid (IFA) received); place of delivery; postnatal care (PNC) for mother and/or newborn within 48 hours of delivery; and early initiation of breastfeeding within an hour of delivery. In addition, we analysed trends at baseline and endline in Caesarean (C-) sections among births in the three years prior to the surveys. The ANC indicators were available for the youngest surviving child born within the three years before DLHS and five years before the NFHS. The place of delivery, PNC, and initiation of breastfeeding were analysed for the last three births in the three years before DLHS and five years before the NFHS. We studied neonatal mortality rates (NMR) for the 10 years prior to the surveys using the birth history data from 2002–2004 DLHS and the pooled NFHS data. The 2007–2008 DLHS was excluded for the NMR estimation, as that survey collected only partial birth histories. We further examined the source of care in the same data, including public and private facilities for all services, public Urban Health and Nutrition Days (UHND, a community outreach platform) for ANC and PNC, and public or private hospitals versus lower-level facilities for delivery care. UHND did not have its own category but was created by combining those who received any ANC or PNC visit at a sub-centre, Anganawadi centre, or Integrated Child Development Service (ICDS) centre, as these are public service locations where UHNDs are usually conducted. We also compared NMR levels by public and private delivery care.

To analyse inequalities, wealth deciles were used instead of quintiles, being more useful for identifying smaller subgroups with markedly lower intervention coverage than the rest of the population. However, wealth quintiles were used wherever the cell sizes were low (Supplementary Table S1a and b). Household wealth deciles or quintiles were computed using principal component analysis consistent with other Demographic and Health Survey (DHS) methods [27], separately for urban households based on household living conditions and assets common in all four surveys.

We analysed trends by computing average annual rates of change (AARC) through simple regression analysis. For measuring inequality trends in intervention coverage, we used the absolute slope index of inequality (SII), relative concentration index (CI) [28], and inequality pattern index (IPI) [29]. SII multiplied by one hundred is interpreted as the percentage point difference in the coverage indicator between the most and least deprived person in the population. The CI measures how much an indicator is concentrated toward the rich or the poor and ranges from −1 to +1. The CI is zero with no wealth-related inequality. The IPI measures the inequality pattern, helping to assess whether observed inequality is mostly attributable to coverage in the richest being markedly higher than the other nine deciles (top pattern of inequality, indicated by more positive values) or due to the poorest lagging compared to the other deciles (bottom inequality, indicated by more negative values).

## Results

### Trends and Inequalities in Urban Neonatal Mortality

**Fig. 1:**
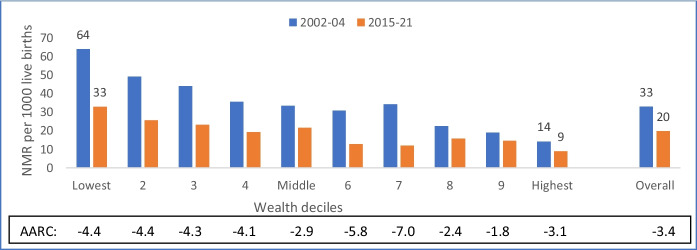
Trends in neonatal mortality in the 10-year period before the surveys by household wealth decile, urban India, 2002–2004 and 2015–2021

The urban NMR among births in the 10 years before the surveys (Fig. 1) declined from 33 to 20 per 1000 live births in 2002–2004 and 2015–2021, respectively (AARC of −3.4%). The decline has been much faster for the lower deciles. For instance, in the lowest decile, the NMR halved from 64 to 33 per 1000 live births during the same time period, with an AARC of −4.4%, compared to the highest decile’s NMR declining from 14 to 9 per 1000 live births, with an AARC of −3.1%. Nevertheless, the absolute difference in NMR between the lowest and highest deciles halved from 50 to 24 units.

### Trends and Inequalities in Maternal and Newborn Health Service Coverage

MNH intervention coverage in urban India at baseline was greater than 75% for receiving any ANC by a skilled provider and for two ANC contents, namely, TT injection and IFA tablets (Table 1). By 2015–2021, coverage increased to over 85% for all seven ANC contents and facility delivery. For the other MNH services, recent coverage levels ranged from 50% for early initiation of breastfeeding to 81% for PNC. The fastest increase was in C-section rates from 16% to 30%, with an AARC of 5%.

**Table 1:** Trends in the coverage of key maternal and newborn health (MNH) services and average annual rates of change in urban India, 2002–2008 and 2015–2021

Indicator	2002–08		2015–21		AARC %
	*% (95% CI)*	*N*	*% (95% CI)*	*N*	
**Received ANC from a skilled provider**	85.8 (85.6–86.1)	94,787	90.2 (89.9–90.5)	85,548	0.42
**Received first ANC in first trimester**	61.0 (60.6–61.5)	93,440	72.2 (71.7–72.7)	85,360	1.40
**Received 4 or more ANC visits**	55.6 (55.2–56.0)	95,281	68.3 (67.8–68.8)	84,678	1.72
**ANC contents:**					
Weight measured Blood pressure measured Blood sample taken Urine sample taken Abdominal examination done TT injection taken IFA tablets received	67.4 (67.1–67.8) 66.9 (66.5–67.2) 69.3 (68.9–69.7) 70.8 (70.4–71.2) 70.3 (69.9–70.6) 85.7 (85.4–85.9) 76.8 (76.4–77.1)	94,787 94,785 94,787 94,785 94,784 94,039 90,677	90.0 (89.7–90.3) 90.2 (89.9–90.4) 89.3 (89.0–89.6) 89.0 (88.7–89.3) 88.6 (88.2–88.9) 88.7 (88.3–89.0) 86.8 (86.5–87.2)	85,548 85,548 85,548 85,548 85,548 85,039 85,478	2.40 2.49 2.12 1.91 1.93 0.29 1.03
**Facility delivery**	70.1 (69.7–70.4)	94,826	91.1 (90.8–91.3)	108,152	2.18
**C-section rate**	16.3 (16.0–16.7)	94,826	30.1 (29.7–30.6)	108,152	4.72
Mother or child received any postnatal care within 48 hours of delivery*	67.9 (67.4–68.4)	40,761	81.2 (80.8–81.6)	85,572	1.63
Initiated breastfeeding within an hour after delivery (vaginal deliveries only)*	45.3 (44.8–45.8)	33,515	49.4 (48.8–49.9)	75,022	0.79

*Baseline coverage excludes 2002–2004 DLHS as the definitions used in that survey for PNC and early initiation of breastfeeding were not consistent with the other surveys.

**Fig. 2:**
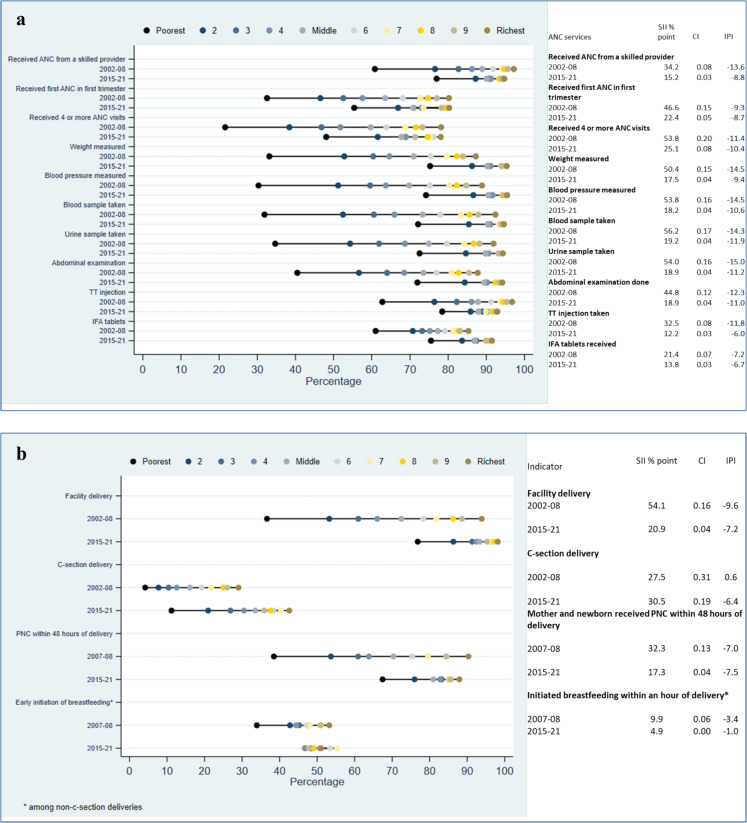
**a** Inequalities in the coverage of antenatal care (ANC) in urban India by household wealth deciles, 2002–2008 and 2015–2021. **b** Inequalities in the coverage of delivery and postnatal care (PNC) services in urban India by household wealth deciles, 2002–2008 and 2015–2021

Wealth-based inequalities also reduced across indicators, as reflected by reductions in the SII and CI between time periods (Fig. 2). For instance, the absolute difference between the richest and poorest deciles in facility delivery more than halved from 57 to 21 percentage points. The lowest deciles likewise had faster coverage improvements as reflected in AARCs (Table S2).

Although the coverage inequalities have notably reduced, visible bottom inequalities persisted for all services. For instance, even in 2015–2021, nearly a 10-percentage point difference in facility delivery coverage remained between deciles 1 (poorest) and 2 (second poorest) (77% versus 86%, respectively), compared to a three or less percentage point difference between the consecutive deciles (91% in decile 3 and 93% in decile 4). The persistence of bottom inequalities is also reflected in the IPI, which was negative for all services; the relative gap improved slightly for ANC and facility delivery but worsened for the C-section rate and PNC coverage. The lowest C-section rate at endline was observed among the poorest decile at 11% (Fig. 2), which was just within the cut-off for ‘met need’ of 10% C-section as per WHO, compared to levels over 20% for the rest of the deciles (suggesting much higher levels of elective C-sections in those groups) [30].

### Comparing Relative Progress Among the Lowest Three Deciles to Understand Heterogeneity Among the Poorest

Comparing the progress specifically among the lowest three wealth deciles and across the indicators may throw additional light on how similar the improvements have been across the poorest urban populations. Slower or no progress in the lowest decile would indicate that despite overall reductions in inequality, there are still segments of the population that are left behind. For this, we compared MNH indicator values of the poorest three deciles in 2015–2021 with the 2002–2008 values across deciles to assess how much they have progressed relative to the other groups (Table 2). For instance, the poorest (D1) had a facility delivery coverage of 77% in 2015–2021, which was between the facility delivery coverage among D5 (72%) and D6 (78%) in 2002–2008. Hence, the relative ‘gain’ for them over time was a shift in 5–1 = 4 ‘deciles’. The greatest ‘gains’ among the lowest three deciles were in NMR (gains of 5–6 deciles) and facility delivery (gains of 4–6 deciles). The smallest ‘gains’ were for ANC and hospital delivery coverage—for which at endline, the lowest three deciles only reached the baseline values of deciles three or less ahead of them.

**Table 2.**
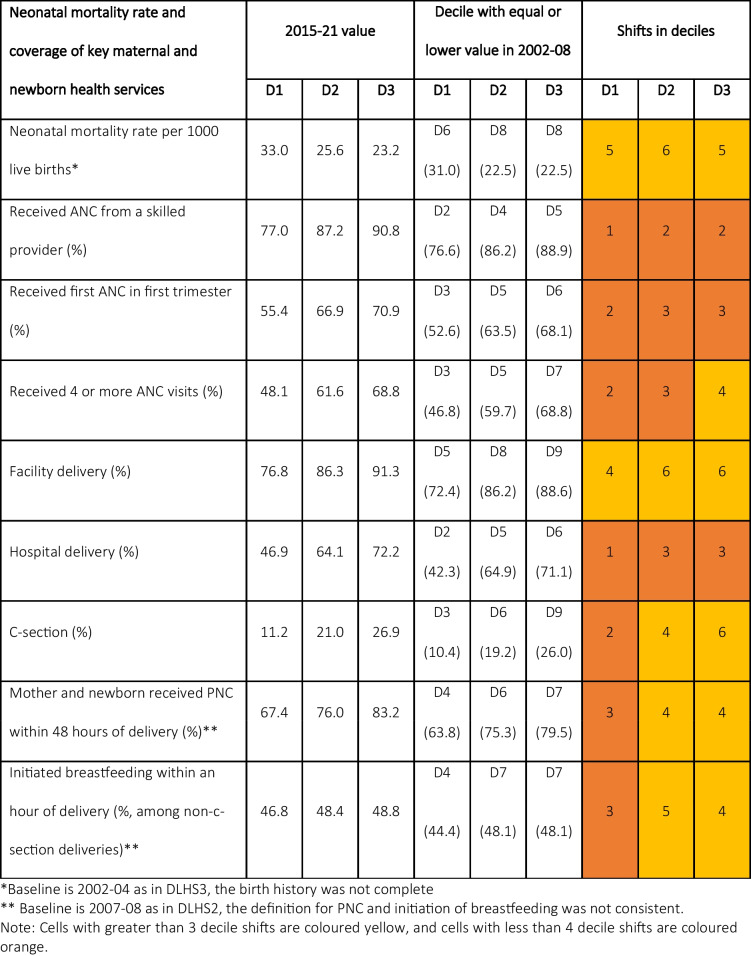
Shifts in neonatal mortality and key maternal and newborn health service coverage indicators among the bottom three deciles between 2002–08 and 2015–21, urban India

### MNH Service Coverage by Source Across Wealth Deciles

**Fig. 3:**
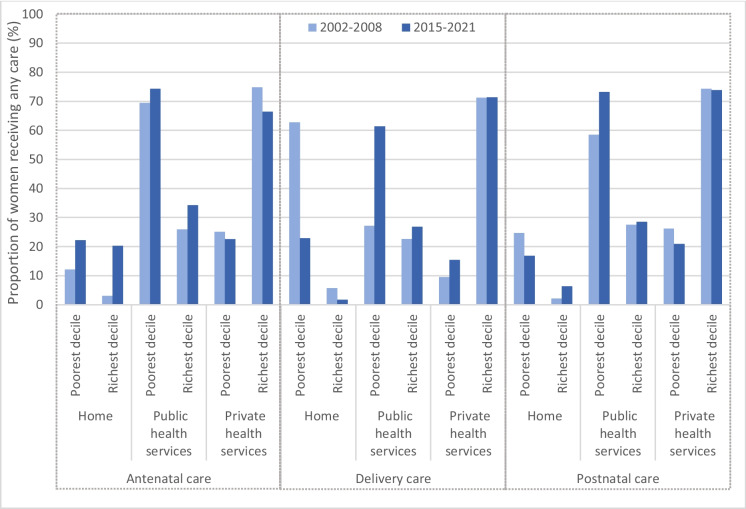
The source of antenatal, delivery, and postnatal care services among the poorest (D1) and richest (D10) deciles, 2002-08 and 2015-21

The public sector’s provision of MNH services has expanded, particularly among the lowest wealth deciles (Fig. 3; Table S3). Accessing ANC at the public UHNDs within the community, as compared to public or private facilities, particularly increased in the lowest decile (from 11% to 23%). ANC was also received more at home, which could be explained by the expansion of Community Health Workers (CHWs) such as Auxiliary Nurse Midwives (ANMs) and Accredited Social Health Activists (ASHAs) making home visits to provide ANC. The use of public health facilities for delivery has increased disproportionately in the poorest wealth decile compared to richer groups: a 14-percentage point increase in case of public hospitals and a 20-percentage point increase for lower-level public facilities. Contrarily, the use of public hospitals for C-sections has been lowest and increased less among the lowest decile than other groups (Table S3). The greatest proportion of women in the poorest decile received PNC from a public health service, increasing from 59% to 73% between periods. Consistent over time, nearly all the richest relied on private facilities for PNC.

### NMR by Source of Delivery Care Among the Poorest and the Richest

**Fig. 4:**
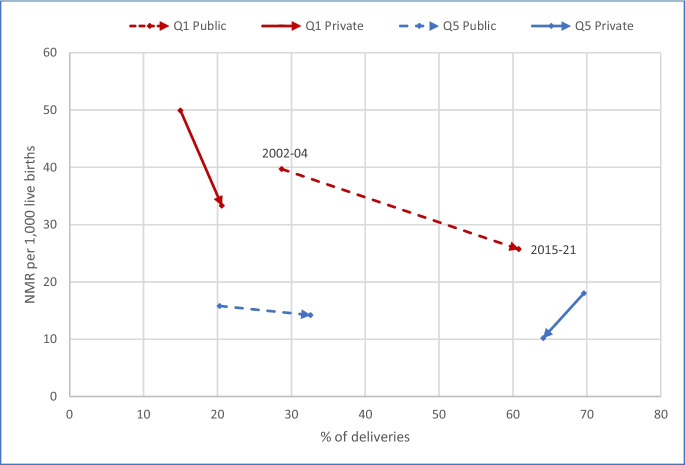
Trends in neonatal mortality rate by source of delivery care (private or public facilities) among the poorest (Q1) and richest (Q5) quintiles, 2002-04 and 2015-21

Deliveries among women in the poorest quintile increased much more and higher in public than private facilities. Meanwhile, they experienced comparably large reductions in NMR in private and public facilities (Fig. 4), from 50 to 33 per 1000 live births versus from 40 to 26 per 1000 live births in 2002-04 and 2015-21, respectively. There was also an increase in births at public facilities among the richest quintile, yet the level of private health facility deliveries was stagnant. Still, they experienced relatively less NMR reduction in public health facilities in this time (from 16 to 14 per 1000 live births) than in private health facilities (18 to 10 per 1000 live births). Unlike for the poorest quintile, the NMR in the richest quintile in private facilities remained lower than in the public health facilities. A large NMR gap between the poorest and richest persisted among the private sector deliveries and, to a lesser extent, public facilities in both periods.

## Discussion

We analysed national surveys to understand trends in coverage of MNH services in urban India overall and by household wealth decile over the last two decades. Coverage substantially increased for most services among births between 2002–2008 and 2015–2021, with a steeper increase among the urban poor compared to richer groups, thus reducing wealth-based inequalities. In particular, coverage increased more at public than private health services, especially among the poorest. NMR among births in the poorest wealth quintile decreased more than in the richest group. NMR was consistently lower among public than private facility deliveries for the poorest but not the richest group. Still, the poorest tenth of the population remains well behind all other groups across MNH indicators.

What could explain the improvements in MNH indicators for the poor compared to the rich in urban India over the past two decades? Our findings indicate that the improvements could be attributable to the NUHM’s, and NHM’s, strong focus on health systems strengthening and implementation of several national health programs in the country’s urban areas [7, 31, 32]. The Mission has strengthened the primary and secondary facilities across cities and towns, with focused outreach such as UHND and community awareness via platforms like Mahila Arogya Samiti (MAS) [7]. The Reproductive, Maternal, Newborn, Child and Adolescent Health (RMNCH+A) strategy implemented in urban areas, as part of NUHM, has focused on addressing the health needs of vulnerable and disadvantaged women and children, prioritising urban slums and migrants, through a ‘continuum of care’ model [33]. According to the Government of India’s analysis of its Management Information System in 2023, the NUHM has covered about 275 million people in 1213 cities or towns [8]. Other researchers note that the NUHM may have expanded its reach through hiring and training program managers within municipal corporations for larger cities and in state departments for smaller cities [25]. Under the National Urban Health Mission (NUHM), existing urban healthcare facilities, including Urban Family Welfare Centres (UFWCs) and Urban Health Posts (UHPs), were upgraded and strengthened as Urban Primary Health Centres (UPHCs) [7]. In addition to augmenting staff and supplies at facilities, NUHM aimed to improve their distribution following facility and slum mapping [6, 7]. For example, by March 2023, the government data showed that 5195 UPHCs were operational, of which three-fourths had minimum staffing and service packages, while 10% were functional 24 × 7 providing comprehensive primary health care services [8]. About 60% to 78% of clinical and paramedical staff and 79% to 82% of the program management staff were in position as against the sanctioned posts in these cities and towns [8].

NUHM aimed to improve outreach to poorer communities through MAS (women’s groups) and urban ASHAs, as well as implement UHNDs to bring services within communities [8, 25]. The specific incentive schemes under the NHM such as Janani Suraksha Yojana (JSY) in 2005 and Janani Suraksha Shishu Karyakaram (JSSK) in 2013 were applied in urban areas too. Another study found that the proportion of urban women that used MNH services who were beneficiaries of government conditional cash transfers (mainly JSY incentives) was highest among the poorest [34]. This was starker in states with lower overall coverage, where higher incentive amounts were universally given without means-testing for use of government services. These schemes likely helped improve access to public services, particularly among poorer populations in part by reducing any out-of-pocket expenditure.

Despite the large improvements in the past two decades, our analyses using wealth deciles also highlighted that the poorest 10% of women are now being left behind for most MNH indicators. This has resulted in visible bottom inequalities, a common but concerning pattern when coverage reaches higher levels [29]. We also suspect there could be substantial unmet need for complex services like C-sections among pockets of people within the poorest decile, which we were not able to fully quantify here. Based on in-depth research in four cities of India, a comprehensive report highlighted the complex forces contributing to worse health outcomes among poorer groups: first, having inadequate and insecure housing, often in notified slums with poor living conditions but also in unlisted slums considered illegal. Those living in these conditions also usually face social exclusion based on caste, religion, gender, or other characteristics, as well as occupational vulnerability often with informal or no employment [35]. Likewise, Nambiar and Mander stress that urban health inequalities are heightened by the informality, invisibility, and illegality marking the experiences of poorer groups in contexts like India with unbridled urbanisation and increasingly privatised health markets [36]. Large numbers of migrants live in informal housing; without formal address proof and other identity documentation, they cannot access their rightful social and health-related entitlements. This reduces access to public MNH care, despite the mandate for universal coverage, and causes a reliance on low-quality, expensive alternative health services, raising the burden of out-of-pocket expenses [35, 36].

There is consistent evidence that people incur particularly high and more inequitable costs for emergency obstetric and newborn care (EmONC) in private than public facilities, as this requires more complex in-patient services than routine deliveries [19, 20]. Our study suggests that while rates are low, C-sections among the poorest were higher in the public sector, which may relate to their lower and less inequitable costs than private facilities [20]. In contrast, one study in Uttar Pradesh and Rajasthan found that a sample of urban poor people relied more on the private sector for C-sections, which suggests that availability of public emergency services is insufficient in those states [19]. Another study found that while out-of-pocket expenses for C-sections was multiple times higher in urban private than public facilities, the poorest spent much less than richer groups at private facilities [20]. This suggests that the poorest groups access different kinds of private facilities, which are less costly but possibly of lower quality, which others have termed as ‘segregated care patterns’ between richer and poorer groups [35]. This may partially explain the relatively higher NMR among the poorest than richer groups, particularly in the private sector. This likely also reflects differential case mixes related to the proportion of obstetric and newborn complications and referrals between sectors and groups. Still, they found that private care was perceived by all wealth groups as having better quality, which led to a preference for those more expensive emergency services. Conversely, all wealth quintiles spent much less and relatively similar amounts on EmONC in public facilities [20]. This reaffirms that enhancing access and quality of public emergency care services is essential to further reduce inequalities in both MNH service coverage and outcomes in urban India.

While we found that the NUHM’s primary care and outreach platforms like UHNDs have increasingly reached poorer communities with MNH services, a couple of implementation research studies have uncovered challenges particular to slum areas that could contribute to ongoing bottom inequalities. A study in Nagpur, Maharashtra, found that UHND and community health worker catchment areas were unclear and continually changing due to in-migration, leading to unserved pockets or duplication of efforts [37]. They also found little to no involvement of MAS or urban local bodies despite NUHM policy supporting this. Those UHNDs focused most on pregnancy registration and IFA tablet distribution, but less on birth preparedness for high-risk pregnancy identification, or any PNC provision, in part because they lacked adequate supplies or drugs and limited privacy for physical exams in spaces where they could meet in slums. Another study in Behrampur, Odisha, found that women in slums attended and received some aspects of ANC at the UHND, but other contents such as weighing and taking blood pressure of pregnant women were also low in part because privacy was hard to achieve [38].

To further reduce bottom inequalities through the NUHM’s intentional policies, it is crucial to identify and overcome the particular challenges for improving MNH service availability, access, affordability, and quality for poorer urban populations. The NUHM has increasingly sought to implement a comprehensive primary health care approach with these goals in mind [6, 8]. The 2023 guidelines re-emphasised making improvements in health worker density, service quality, and referral networks to reduce OOPE and improve outcomes in pursuit of universal health coverage [8]. Relatedly, others have recommended increasing government financing, especially for primary care and health insurance mechanisms beyond curative services, better coordinated partnerships and governance mechanisms across private and public sectors, and between municipal and state levels, echoing the 2023 framework [8, 18, 25, 35]. Many have promoted strengthening comprehensive PHC with a focus on health equity along similar lines as the NUHM, not only by enhancing multilevel governance and access to affordable and quality health services but ensuring community engagement and intersectoral action involving education, urban planning, WASH, and other areas to address health vulnerabilities amidst the complexity of each urban context [18, 25].

More attention may be needed to the underlying principles of targeting, and particularly affirmative schemes and incentives, to optimally improve access, affordability, and quality of MNH services among the poorest groups in urban India going forward. Proponents of a proportionate universalism approach for addressing health inequities have suggested that targeting using a *means* approach can often actually maintain or worsen inequalities [39]. By requiring identification or other proof of eligibility to access public schemes and services, means testing or verification could unintentionally feed into the bottom inequalities that still exist in MNH outcomes in urban India. Instead, shifting focus on the *needs* of poorer groups could help better target and tailor programs to address the barriers they face by virtue of who and where they are through ‘positive selectivism’. This means that within universal health policies, particularist approaches to targeting should be developed at a local governance level through sustained community engagement to make public services more responsive to the needs and contexts of poorer populations. Researchers and policy-makers should therefore strive for more contextualised understandings of the heterogeneous and dynamic urban settings in which the poorest groups live that affect their health and healthcare access [18]. This will help to develop and then continually evaluate and optimise strategies under NUHM to target the most vulnerable groups by addressing their particular MNH needs, in order to reduce lingering bottom inequalities and improve outcomes for the poorest women and children living in urban India [35].

The study has implications for tailoring existing policies to better reach the urban poorest. First, needs-based affirmative approaches can be developed in partnership with community-based or non-governmental organisations who work directly with and in these communities. This could involve government contracts for community groups working in slums or with other disadvantaged urban groups to co-develop and deliver bottom-up approaches that make MNH service delivery models more available and acceptable. Second, NUHM strategies must be better tailored to the urban contexts of the poorest rather than applied the same way universally. For example, while UHND service provision is modelled on the Village Health and Nutrition Days (VHNDs), the contexts of rural villages and urban slums are very different. UHNDs could be redesigned to be offered flexibly for slums, in spaces or at times when more people are more available and comfortable. A final recommendation would be to ensure that high-quality EmONC is more accessible to the poorest. This includes addressing high out-of-pocket expenditure for C-sections, starting by assessing whether existing schemes are reaching these groups. ‘Cashless’ C-sections are meant to be provided at public as well as empaneled private facilities when needed under the Pradhan Mantri Jan Arogya Yojana (PM-JAY), a national insurance scheme put in place in 2018 under the Ayushman Bharat program [40]. At the system level, implementation of the scheme must intentionally include facilities where the poorest are more likely to go and ensure that coverage is not hindered by administrative barriers, such as burdensome procedures to sign up. At the service delivery level, during antenatal care, if the provider recognizes that their patient faces financial barriers, they should support them on where and how to access ‘cashless’ delivery care and C-sections if required. This could reduce delays to or between facilities in the case of emergencies. Ultimately, a multi-pronged, contextualised approach would help improve the accessibility, acceptability, and quality of services for the urban poorest groups in India.

The study had some limitations. While larger observed increases in MNH coverage and outcomes alongside reductions in inequalities at public services seem consistent with the NUHM policies, this study cannot directly explain the processes by which NUHM may have played a role, which deserves further study. Analysing wealth using deciles to detect bottom inequalities required pooling the 2002–2008 and 2015–21 survey years to ensure sufficient samples. However, this also made the time points less distinct than before and after the NUHM as they could be without pooling. The results reflect a picture of urban India overall, but it was beyond the study’s scope to compare different city types or sizes, and states or regions, to reflect more diversity.

## Conclusion

Based on large, representative survey data from the last two decades, India has made great strides in reducing wealth-based inequalities in maternal and newborn health coverage and outcomes in urban settings. The greatest improvements were in coverage of public health services. While we cannot directly attribute the results to the changes under NUHM in this study, the positive increases in coverage of the urban poor for MNH indicators studied in the public sector seem related to the augmentation of primary and secondary health infrastructure and implementation of the RMNCH+A strategy under NUHM, in addition to the contextual changes over this period. Rapid improvements with reduced inequalities may not only reflect increased access to public facilities but also point towards improving health education and information among the urban poor, addressing social determinants, and strengthening intersectoral convergence. Yet lingering bottom inequalities still exist, such that the poorest tenth is being left behind on many indicators, and neonatal mortality has not improved as greatly as access to services among them. The results suggest that inequalities can continue to narrow if concerted efforts target and address health system and contextual issues causing bottom inequalities across diverse locales. There is great potential to do so by harnessing NUHM’s comprehensive primary health care policies to develop more localised needs-based approaches, to ensure that even the poorest women and infants can reach affordable, high-quality MNH health services and no longer experience the undue burden of morbidity and mortality in urban India.

## Supplementary Information

Below is the link to the electronic supplementary material.

ESM1 (DOCX 54.9 KB)

## Data Availability

The data analysed in this study are publicly available upon request from the International Institute for Population Sciences, Mumbai (https://www.iipsdata.ac.in/data_catalog).
