# Supplementary material for: The Role of Public Health Services in Reducing Maternal and Newborn Health Inequalities in Urban India: A Survey Analysis of 200,000 Births Over Two Decades
Source: J Urban Health. 2026 Jun 11;103(3):574–86. doi: 10.1007/s11524-026-01062-6 (PMC13315426; doi:10.1007/s11524-026-01062-6)
Supplement: Supplementary file 1 — (DOCX 54.9 KB) [file 11524_2026_1062_MOESM1_ESM.docx]

Supplementary materials

***Table S1a*** *Sample size (numerator and denominator, unweighted) for estimating coverage indicators overall and in each household wealth decile and overall for urban India, 2002-08 and 2015-21*

| **Year/categories** | **Household wealth deciles** | | | | | | | | | |  |
| --- | --- | --- | --- | --- | --- | --- | --- | --- | --- | --- | --- |
|  | **Lowest** | **2** | **3** | **4** | **Middle** | **6** | **7** | **8** | **9** | **Highest** | **Overall** |
| **Denominators for estimating coverage indicators: number of live births in reference period** | | | | | | | | | | | |
| **2002-08** | 8707 | 8181 | 7931 | 7901 | 7588 | 8455 | 5515 | 6997 | 7408 | 7122 | 75805 |
| **Place of delivery** | | | | | | | | | | | |
| Home | 4979 | 3429 | 2707 | 2299 | 1831 | 1608 | 860 | 854 | 761 | 377 | 19705 |
| Public facility | 2288 | 2750 | 2880 | 2788 | 2619 | 2722 | 1597 | 1910 | 1792 | 1560 | 22906 |
| Private facility | 833 | 1391 | 1615 | 2022 | 2419 | 3250 | 2404 | 3469 | 3967 | 4341 | 25711 |
| **2015-21** | 17376 | 13921 | 11047 | 10885 | 8760 | 13836 | 4762 | 8930 | 7851 | 8258 | 105626 |
| **Place of delivery** | | | | | | | | | | | |
| Home | 4216 | 1958 | 1084 | 933 | 640 | 686 | 176 | 321 | 373 | 165 | 10552 |
| Public facility | 10761 | 8696 | 6656 | 5966 | 4706 | 6513 | 2269 | 4222 | 3394 | 2740 | 55923 |
| Private facility | 2360 | 3223 | 3283 | 3950 | 3394 | 6611 | 2300 | 4379 | 4069 | 5330 | 38899 |
| **Denominators for estimating mortality: number of live births in the past 10 years period preceding the survey** | | | | | | | | | | | |
| **2002-04** | 7258 | 6444 | 6449 | 6417 | 6055 | 7170 | 4982 | 5876 | 6873 | 7717 | 65241 |
| **Place of delivery** | | | | | | | | | | | |
| Home | 3889 | 2487 | 1916 | 1665 | 1308 | 1233 | 711 | 734 | 669 | 370 | 14982 |
| Public facility | 1328 | 1593 | 1699 | 1571 | 1456 | 1540 | 943 | 1098 | 1090 | 1213 | 13531 |
| Private facility | 538 | 823 | 1016 | 1203 | 1353 | 1898 | 1479 | 1819 | 2545 | 3164 | 15838 |
| **2015-21** | 36620 | 29166 | 23107 | 23051 | 18829 | 28213 | 10746 | 17817 | 16833 | 17716 | 222098 |
| **Place of delivery** | | | | | | | | | | | |
| Home | 4216 | 1958 | 1084 | 933 | 640 | 686 | 176 | 321 | 373 | 165 | 10552 |
| Public facility | 10761 | 8696 | 6656 | 5966 | 4706 | 6513 | 2269 | 4222 | 3394 | 2740 | 55923 |
| Private facility | 2360 | 3223 | 3283 | 3950 | 3394 | 6611 | 2300 | 4379 | 4069 | 5330 | 38899 |

***Table S1b*** *Sample size (numerator and denominator, unweighted) for estimating mortality overall and in each household wealth quintile and overall for urban India, 2002-08 and 2015-21*

| **Years/categories** | **Household wealth quintiles** | | | | | **Total** |
| --- | --- | --- | --- | --- | --- | --- |
|  | **Lowest** | **2** | **Middle** | **4** | **Highest** |  |
| **Denominators for estimating mortality: number of live births in the past 10 years period preceding the survey** | | | | | | |
| **2002-04** | 13702 | 12866 | 13225 | 10858 | 14590 | 65241 |
| **Place of delivery** | | | | | | |
| Home | 6376 | 3581 | 2541 | 1445 | 1039 | 14982 |
| Public facility | 2921 | 3270 | 2996 | 2041 | 2303 | 13531 |
| Private facility | 1361 | 2219 | 3251 | 3298 | 5709 | 15838 |
| **2015-21** | 16888 | 15832 | 16043 | 12512 | 14530 | 75805 |
| **Place of delivery** |  |  |  |  |  |  |
| Home | 6174 | 2017 | 1326 | 497 | 538 | 10552 |
| Public facility | 19457 | 12622 | 11219 | 6491 | 6134 | 55923 |
| Private facility | 5583 | 7233 | 10005 | 6679 | 9399 | 38899 |
| **Numerators for estimating mortality: number of neonatal deaths in the past 10 years period preceding the survey** | | | | | | |
| **2002-04** | 1218 | 730 | 604 | 408 | 380 | 3340 |
| **Place of delivery** | | | | | | |
| Home | 706 | 281 | 163 | 74 | 45 | 1269 |
| Public facility | 173 | 161 | 143 | 72 | 65 | 614 |
| Private facility | 125 | 133 | 156 | 143 | 163 | 720 |
| **2015-21** | 3029 | 1540 | 1329 | 658 | 700 | 7256 |
| **Place of delivery** | | | | | | |
| Home | 330 | 107 | 59 | 17* | 22* | 535 |
| Public facility | 748 | 334 | 319 | 133 | 147 | 1681 |
| Private facility | 257 | 226 | 226 | 136 | 152 | 997 |
| *Cell size <30 | | | | | | |

***Table S2*** *Average annual rate of change (AARC) in the coverage of key maternal and newborn health (MNH) services by household wealth deciles and overall in urban India during 2002-08 to 2015-21*

| **Indicator** | **AARCs by Household wealth deciles: 2002-08 to 2015-21** | | | | | | | | | |
| --- | --- | --- | --- | --- | --- | --- | --- | --- | --- | --- |
|  | **Lowest** | **2** | **3** | **4** | **Middle** | **6** | **7** | **8** | **9** | **Highest** |
| **Received ANC from a skilled provider** | 1.81 | 1.00 | 0.72 | 0.43 | 0.10 | 0.22 | -0.04 | -0.11 | -0.13 | -0.20 |
| **Received first ANC in first trimester** | 4.09 | 2.79 | 2.31 | 1.83 | 0.83 | 1.16 | 0.08 | 0.46 | 0.12 | 0.01 |
| **Received 4 or more ANC visits** | 6.16 | 3.63 | 2.96 | 2.20 | 0.97 | 1.35 | 0.69 | 0.32 | -0.20 | -0.01 |
| **ANC content**  Weight measured  Blood pressure measured  Blood sample taken  Urine sample taken  Abdominal examination  TT injection taken  IFA tablets received | 6.30  6.88  6.27  5.67  4.42  1.71  1.64 | 3.77  4.04  3.74  3.41  3.06  0.90  1.29 | 3.11  3.25  3.08  2.86  2.61  0.60  1.37 | 2.64  2.80  2.48  2.13  2.12  0.36  1.11 | 1.85  2.00  1.61  1.40  1.51  0.01  0.91 | 1.77  1.80  1.48  1.31  1.52  0.02  1.05 | 1.26  1.23  0.85  0.78  1.03  -0.32  0.76 | 1.04  1.04  0.70  0.56  0.86  -0.23  0.68 | 0.87  0.84  0.51  0.42  0.68  -0.24  0.61 | 0.68  0.55  0.18  0.20  0.55  -0.31  0.54 |
| **Facility delivery** | 5.69 | 3.72 | 3.11 | 2.59 | 1.95 | 1.57 | 1.28 | 0.92 | 0.55 | 0.34 |
| **C-section delivery** | 7.57 | 7.66 | 7.30 | 6.81 | 5.64 | 5.31 | 4.75 | 3.13 | 2.46 | 2.95 |
| **Mother or child received any postnatal care within 48 hours of delivery** | 5.10 | 3.16 | 2.83 | 2.37 | 1.28 | 1.10 | 0.63 | 0.10 | 0.13 | -0.25 |
| **Initiated breastfeeding within an hour after delivery (vaginal deliveries only)** | 2.92 | 1.13 | 0.70 | 0.47 | 0.57 | 1.13 | 1.27 | -0.38 | -0.56 | -0.39 |

***Table S3*** *The source of key maternal and newborn health (MNH) service provision (percentage of women receiving any care from each source), and average annual rates of change in urban India, overall and by household wealth deciles, 2002-08 and 2015-21*

| **Source of service** | **Household wealth deciles** | | | | | | | | | | **Overall** | **SII % point** | **CI** | **IPI** |
| --- | --- | --- | --- | --- | --- | --- | --- | --- | --- | --- | --- | --- | --- | --- |
|  | **Poorest** | **2** | **3** | **4** | **Middle** | **6** | **7** | **8** | **9** | **Richest** |  |  |  |  |
| **ANC** | | | | | | | | | | | | | |  |
| **2002-08**  Home  Public health service  Public facility  Public UHND  Private health service | 12.1  69.4  60.8  10.5  25.1 | 8.8  67.2  60.7  8.9  32.9 | 7.1  64.4  58.4  7.9  37.0 | 6.3  59.6  55.2  6.1  43.1 | 5.1  53.8  49.8  5.4  48.9 | 5.1  47.5  44.4  4.3  54.8 | 4.6  42.2  40.0  3.0  59.9 | 3.3  36.5  35.6  1.6  65.1 | 3.1  32.9  31.6  1.7  68.6 | 3.0  25.9  25.4  0.8  74.8 | 5.8  50.2  46.4  5.0  50.9 | -8.3  -46.6  -10.5  49.7 | -0.19  -0.15  -0.14  -0.29  0.17 | 3.41  -5.16  -6.67  1.22  -1.96 |
| **2015-21**  Home  Public health facility/ provider  Public facility  Public UHND  Private health facility/ provider | 22.2  74.3  57.8  28.2  22.5 | 23.1  67.8  55.9  21.1  32.1 | 23.0  64.1  54.3  17.5  35.7 | 21.7  60.0  51.2  16.0  42.0 | 23.1  56.6  49.2  15.1  42.8 | 19.9  48.8  43.0  12.0  52.8 | 22.9  52.7  45.2  15.3  51.1 | 19.3  50.8  44.9  12.3  52.1 | 19.1  47.7  40.5  13.6  57.2 | 20.3  34.2  29.0  8.2  66.4 | 21.4  56.2  47.5  16.0  44.8 | -3.8  -34.5  -16.1  39.5 | -0.00  -0.09  -0.08  -0.13  0.17 | -0.37  -3.96  -8.37  4.34  -0.65 |
| **Delivery** | | | | | | | | | | | | | |  |
| **2002-08**  Home  Facility  Public health facility  Private health facility  Hospital  Public hospital  Private hospital  Lower-level health facility  Public lower-level facility  Private lower-level facility  **2015-21**  Home  Facility  Public facility  Private facility  Hospital  Public hospital  Private hospital  Lower-level health facility  Public lower-level facility  Private lower-level facility | 62.8  36.7  27.1  9.5  26.3  17.0  9.3  10.4  10.2  0.2  22.9  76.8  61.4  15.4  46.9  32.1  14.8  29.9  29.3  0.6 | 46.1  53.2  35.2  18.1  42.3  24.7  17.7  10.9  10.5  0.4  13.5  86.3  60.1  26.2  64.1  38.5  25.6  22.2  21.6  0.6 | 38.4  61.0  39.2  21.8  51.3  29.9  21.3  9.7  9.3  0.5  8.5  91.3  57.4  33.9  72.2  39.0  33.2  19.1  18.4  0.6 | 33.5  66.0  37.7  28.4  57.4  29.6  27.9  8.6  8.1  0.5  7.2  92.5  53.0  39.5  75.3  36.5  38.8  17.2  16.5  0.8 | 27.0  72.4  36.2  36.3  64.9  29.1  35.8  7.6  7.1  0.5  6.5  93.3  51.7  41.6  77.9  37.1  40.8  15.4  14.6  0.8 | 21.0  78.2  35.1  43.1  71.1  28.8  42.2  7.2  6.3  0.9  3.9  96.0  41.3  54.7  83.1  29.2  53.9  12.9  12.1  0.8 | 17.4  81.8  30.9  50.9  75.6  25.8  49.9  6.1  5.1  1.0  3.2  96.5  43.9  52.6  81.8  30.1  51.7  14.7  13.8  0.9 | 13.4  86.2  28.8  57.4  80.9  24.5  56.4  5.4  4.4  1.0  2.7  97.2  42.7  54.5  83.7  29.8  53.9  13.5  12.8  0.7 | 10.8  88.6  26.7  61.9  82.7  21.9  60.8  6.0  4.8  1.1  4.6  95.2  38.5  56.8  82.4  26.6  55.9  12.8  11.9  0.9 | 5.7  93.9  22.6  71.2  88.4  18.6  69.8  5.4  4.1  1.4  1.7  98.1  26.8  71.4  89.6  19.2  70.4  8.6  7.5  1.0 | 29.3  70.1  32.4  37.7  62.1  25.2  37.0  8.0  7.2  0.7  8.4  91.1  49.2  41.9  73.6  32.4  41.2  17.5  16.7  0.7 | -54.1  54.1  -8.2  61.9  59.5  -0.6  60.9  -6.4  -7.6  1.2  -20.8  20.9  -32.6  51.1  38.4  -13.8  50.8  -19.0  -19.4  0.3 | -0.31  0.16  -0.01  0.31  0.19  0.02  0.31  -0.07  -0.12  0.33  -0.38  0.04  -0.09  0.23  0.10  -0.04  0.24  -0.17  -0.18  0.11 | 9.90  -9.64  -15.05  5.39  -9.55  -14.80  5.24  -0.09  -0.25  0.15  7.80  -7.18  -10.17  2.99  -10.74  -13.58  2.84  3.56  3.41  0.15 |
| **C-section delivery** | | | | | | | | | | | | | |  |
| **2002-08**  Public facility  Private facility  **2015-21**  Public facility  Private facility | 2.5  1.6  6.4  4.8 | 4.0  3.7  11.7  9.3 | 5.1  5.3  12.1  14.8 | 5.1  7.5  12.3  18.2 | 6.4  9.7  12.8  20.7 | 6.1  13.0  10.8  27.6 | 5.5  16.0  11.8  28.5 | 6.0  18.9  11.2  26.4 | 5.0  20.8  8.9  27.0 | 4.4  24.4  7.0  35.6 | 5.0  11.3  10.5  19.9 | 2.2  25.8  0.2  30.7 | 0.12  0.40  0.04  0.28 | -3.10  -3.40  -7.60  -0.60 |
| **PNC*** | | | | | | | | | | | | | |  |
| **2008**  Home  Public health service  UHND  Private health service  **2015-21**  Home  Public health service  UHND  Private health service | 24.7  58.5  0.8  26.2  16.9  73.2  4.7  20.9 | 17.7  58.2  0.8  33.0  15.0  66.8  3.0  31.1 | 15.2  57.6  0.5  34.5  11.3  62.8  2.8  37.8 | 12.5  53.0  0.3  41.2  11.3  57.2  1.8  44.7 | 9.6  49.8  0.2  47.2  10.0  55.5  2.1  46.3 | 7.4  44.7  0.3  54.3  7.5  44.8  1.7  58.4 | 5.9  39.7  0.2  59.5  10.1  46.7  2.6  56.6 | 4.5  35.4  0.1  64.9  8.7  44.6  1.1  58.4 | 4.1  32.5  0.1  68.2  9.3  41.3  1.6  60.5 | 2.1  27.5  0.1  74.3  6.3  28.5  0.9  73.8 | 9.8  45.4  0.3  51.3  10.6  52.7  2.2  48.6 | -21.3  -35.3  -0.7  49.3  -9.3  -40.6  -0.3  47.0 | -0.35  -0.14  -0.40  0.17  -0.12  -0.12  -0.18  0.19 | 7.17  -4.82  0.24  -2.30  2.12  -3.60  1.16  -2.39 |
| *Excludes DLHS-2.  Notes: (1) The percentages by source of ANC do not add to 100% because multiple sources were recorded in the surveys (2) The NFHS 5 collected information on place of PNC checkup only for mothers who did not receive any PNC checkup before discharged from health facility or those who delivered at home, whereas, NFHS 4 collected place of PNC for mothers while they remained in a health facility post-delivery and the place of health check for newborn is collected for all children regardless of their place of birth. (2) DLHS 3 collected place of PNC checkup separately for all mothers and newborn regardless of place of delivery. | | | | | | | | | | | | | | |
